# Extracellular embryo genomic DNA and its potential for genotyping applications

**DOI:** 10.4155/fso.15.62

**Published:** 2015-11-01

**Authors:** Luca Galluzzi, Simone Palini, Silvia De Stefani, Francesca Andreoni, Mariangela Primiterra, Aurora Diotallevi, Carlo Bulletti, Mauro Magnani

**Affiliations:** 1 Department of Biomolecular Sciences, University of Urbino ‘Carlo Bo’, 61029 Urbino (PU), Italy; 2 IVF Unit, ‘Cervesi’ Hospital Cattolica, 47841 Cattolica (RN), Italy

**Keywords:** blastocoele fluid, culture medium, gDNA, human embryos, *MTHFR*

## Abstract

**Background:**

Preimplantation genetic diagnosis (PGD) currently relies on biopsy of one or few embryo cells. Our aim was to evaluate the embryo extracellular matrices (spent medium and blastocoele fluid) as source of DNA for embryo genotyping.

**Results/methodology:**

We first evaluated the amplifiability and the amount of genomic DNA in spent embryo culture media from day 3 (n = 32) and day 5/6 (n = 54). Secondly, we evaluated the possibility to genotype the *MTHFR* polymorphism C677T from media at day 5/6 (n = 8) and blastocoele fluids (n = 9) by direct sequencing. The C677T polymorphism detection rate was 62.5 and 44.4% in medium and fluid, respectively.

**Conclusion:**

A noninvasive approach for embryo genotyping was possible, but still with limitations due to low detection rate and possible allele dropout.

## Abstract (lay abstract)

PGD currently relies on biopsy of embryo cells, which could imply some risk of embryo damage. Since embryo DNA was retrieved both in blastocoele cavity and culture medium, we evaluated if this extracellular DNA could be useful to obtain medical-related genetic information from the embryo. First, we used multicopy genes to verify amplifiability and amount of DNA in medium, then we amplified and sequenced one gene fragment containing a polymorphism of medical importance in a subset of samples. The polymorphism detection rate was not yet high enough to warrant clinical application but we demonstrated that this approach was possible.

**Figure 1.**
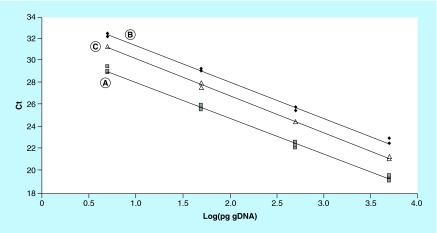
**Representative standard curves constructed with 1:10 scalar dilutions (5000 to 5 pg per reaction tube) of purified human genomic DNA.** Curve **(A)** (Y = -3.24X + 31.32; R2 = 0.996) was constructed with human gDNA only; curve **(B)** (Y = -3.27X + 34.69; R2 = 0.995) and curve **(C)** (Y = -3.34X + 33.59; R2 = 0.998) were constructed with the addition of 2 µl of medium G-1 PLUS and G-2 PLUS, respectively. Ct: Threshold cycle; gDNA: Genomic DNA.

The preimplantation genetic diagnosis (PGD) of a multitude of hereditary genetic disorders currently relies on invasive biopsy procedures [1]. However, cleavage stage biopsy (day 3) in particular has been associated with a small but significant risk of embryo's damage [2–4].

In this context, it would be intriguing to investigate genetic content of embryo matrices obtained with noninvasive or low-invasive procedures. Recently, it has been shown that genomic DNA (gDNA) can be detected in the blastocoele of the human embryo, and that this DNA could be suitable for PCR, whole-genome amplification (WGA) and array Comparative Genomic Hybridization (aCGH) [5]. Successively, the possibility to perform preimplantation genetic screening (PGS) on gDNA from blastocoele fluid was also investigated [6]. Moreover, in the veterinary field, the gDNA was also detected in blastocoele fluid from equine embryos [7]. Genomic and mitochondrial DNA (mtDNA) was also detected in the secretome of human cleavage-stage embryos in day 3 culture medium [8]. In particular, mtDNA in spent day 3 medium has been evaluated as marker of blastocyst potential and implantation outcome [9]. However, no studies have been performed to evaluate the potential of these noninvasive matrices as a source of DNA suitable for genotyping.

In this work, we first evaluated the presence, amplifiability and the amount of gDNA in spent culture media from embryos at different developmental stages (cleavage stage [day 3], and blastocyst stage [day 5/6]) by performing quantitative PCR on two multicopy genes (*TBC1D3* and *TSPY1* located on chromosome 17 and Y, respectively). Multicopy genes were chosen to increase chances of amplification; moreover, the target gene on Y chromosome was chosen to further evaluate the reliability of PCR results in pregnancies or by comparison with expected male/female ratio. Then, as a proof of concept about the possibility to detect a point mutation in gDNA from blastocoele fluid or day 5/6 spent culture media samples, we attempted to genotype the *MTHFR* C677T (rs1801133) polymorphism [10] in samples from blastocysts derived from IVF patients known to carry the 677T mutation.

## Methods

### Embryo culture & sample collection

Human fertilized oocytes obtained from patients carrying the *MTHFR* 677T allele were individually cultured until day 3 in 10 μl of G-1 PLUS medium (Vitrolife), and from day 3 to days 5/6, in 10 μl of G-2 PLUS medium (Vitrolife). Medium droplets were overlaid with paraffin oil (ovoil, vitrolife) and incubated at 37°C in a humidified atmosphere of 6% CO_2_ and 5% O_2_. Spent embryo culture media were collected in PCR tubes after embryo removal. Appropriate precautions were taken to prevent contamination (i.e., sterile, PCR-grade tips and tubes) and sample manipulation was performed under a sterile hood by laboratory personnel wearing gloves and coat. Moreover, medium droplets incubated without embryos were also processed for PCR negative controls. A total of 86 samples (32 spent media from day 3 and 54 from day 5/6) were used in the present study. Blastocoele fluids were collected before vitrification on day 5 or 6, from nine supernumerary embryos as previously described [5,11]. Trophectoderm biopsy was performed by removing three to five cells that had herniated through the zona pellucida from five embryos. The procedure was carried out in G-Gamete medium (Vitrolife). The retrieved cells were transferred to a PCR tube containing 5 µl of Tris-EDTA pH 8. Before trophectoderm biopsy and blastocoele fluid collection, the embryos were transferred to fresh medium, to avoid accidental gDNA release in the medium during these procedures. All samples were immediately frozen and stored at -80°C until processing.

### Detection & quantification of gDNA in embryo culture media

gDNA amplifiability was directly evaluated in 86 spent embryo culture media by quantitative PCR on the multicopy gene *TBC1D3* as described previously [5], with slight modifications. In particular, *TBC1D3* primer concentration was reduced to 100 nM and 2 μl of medium was added directly to 25 μl reaction mixture as source of DNA template. A nontemplate control was included in each PCR run. Moreover, negative controls consisting of empty culture droplets were also tested for *TBC1D3* amplification to exclude contamination. gDNA quantification was performed using a standard curve constructed with human gDNA from 5000 to 5 pg per reaction. Two μl of G-1 PLUS medium or G-2 PLUS medium were also added to each reaction tube to take into account the inhibitory effects of medium at day 3 or day 5/6, respectively. *TSPY1* amplification was performed as described [5] to assess the presence of the Y chromosome.

### Whole-genome amplification

The gDNA was amplified from 2.5 μl of culture media, 2.5 μl of biopsy samples and 4 μl of blastocoele fluid samples using PicoPLEX WGA kit (Rubicon Genomics, MI, USA) according to manufacturer's procedure. Positive and negative (no template) controls were also run to confirm DNA amplification and check for accidental contamination, respectively. The amplified gDNA (referred to hereafter as WGA DNA) was used as template in downstream PCR reactions without further purifications.

### Detection of *MTHFR* C677T polymorphism

A 109 bp-fragment of *MTHFR* gene containing the C677T (rs1801133) polymorphism was amplified from 2 μl of WGA DNA diluted 1:10 using primers MTHFR C677T_F (5′-CGAAGCAGGGAGCTTTGAGG-3′) and MTHFR C677T_R (5′-GCGGAAGAATGTGTCAGCCT-3′). The PCR was performed in a final volume of 50 μl, using 100 nM of each primer and 1 unit of Hot-Rescue DNA Polymerase (Diatheva srl), with the following conditions: 94°C for 10 min, 40 cycles at 94°C for 15 s, 56°C for 15 s and 72°C for 10 s. The PCR products were separated on 2% agarose gel, extracted using QIAquick Gel Extraction Kit (Qiagen), and directly sequenced. Sequencing was performed in both directions with the BigDye Terminator Cycle Sequencing Kit and run in the ABI PRISM 310 Genetic Analyzer (Applied Biosystems).

### Statistical analysis

Statistical analysis was performed with GraphPad Prism 5 software (GraphPad Software, CA, USA).

## Results

### Evaluation of gDNA in embryo culture media

The gDNA in spent medium samples was detected and quantified by real-time PCR using the multicopy gene *TBC1D3* as target. Amplifiable DNA was found in 93.7% and 94.4% of spent media on day 3 and day 5/6, respectively (Table 1).

The gDNA content in the media was estimated using a standard curve constructed with purified human gDNA ranging from 5000 to 5 pg per reaction (each reaction tube was run in triplicate). To evaluate the potential inhibitory effects of medium components on PCR reactions, in addition to the standard curve generated with template DNA only (curve A), other curves were generated by adding 2 μl/tube of medium G-1 PLUS (curve B) and G-2 PLUS (curve C). The presence of media significantly delayed the Ct values in curves B and C, while the reaction efficiencies remained similar among all curves (99–103%) (Figure 1). Therefore, standard curves B and C were used to estimate DNA amount in medium at day 3 and 5/6, respectively. Based on these curves, we estimated that 80 ± 70 pg (range 3–257 pg; median 58 pg) and 99 ± 113 pg (range 2.1–633 pg; median 67 pg) of gDNA were present in the medium on day 3 and day 5/6, respectively. The calculated DNA amounts did not differ significantly between the two culture media (Mann–Whitney test; p > 0.05).

The same samples were also tested for the amplification of *TSPY1* gene (located on Y chromosome). In spent media at day 3, the *TSPY1* gene was amplified in only 2 samples out of 32 (6.7%), while in spent media at day 5/6, it was amplified in 21 samples out of 54 (41.2%), evidencing that the gene TSPY1 appears poorly amplifiable before day 5/6. Notably, sex determination in five medium samples at day 5/6 was confirmed on corresponding pregnancies.

Moreover, analysis of different media from the same embryos (n = 16) confirmed that *TSPY1* amplification can be performed more reliably at day 5/6 (33% male) respect to day 3 (6.2% male) (Table 2). The amplification results from medium at day 5/6 was also confirmed in two embryos (B1 and B4) from trophectoderm biopsied cells.

### Detection of *MTHFR* C677T polymorphism from embryo media & from blastocoele fluid

The *MTHFR* amplification performed in a subset of samples not subjected to WGA was not successful (not shown). Therefore, the *MTHFR* C677T polymorphism was investigated in WGA DNA from embryo culture media and blastocoele fluid, in the attempt to show the possibility to detect point mutations from noninvasive matrices. In light of the results stated above, medium at day 5/6 was selected as more representative of the embryo genome, and it was used as source of DNA to genotype the *MTHFR* C677T polymorphism. To this end, WGA was performed to increase chances to amplify single copy gene. The *MTHFR* fragment amplification was attempted in eight WGA samples, then the PCR product was purified and sequenced in both directions to monitor C677T polymorphism. The amplification and genotyping was successful in five out of eight medium samples (62.5%) (Table 3). Trophectoderm biopsies were available for embyos 1IB, 3IB, 4IB. In embryo 3IB the genotyping result was confirmed on the corresponding trophectoderm biopsy; in another case (embryo 1IB) the biopsy showed an heterozygous genotype (C/T) while in medium sample only one allele (C) was amplified, accounting for a possible allele dropout. Finally, in embryo 4IB, the *MTHFR* amplification from medium was not successful.

WGA was also performed on blastocoele fluid from nine embryos. PCR was then performed to amplify a *MTHFR* fragment, and the C677T polymorphism was genotyped as described above. The amplification was successful in four out of nine fluid samples (44.4%); in one case (embryo 3IB) the trophectoderm biopsy was available and the genotype was confirmed (Table 4).

## Discussion

PGD is used during an assisted reproduction treatment to select genetically normal embryos in case of couples with high risk of transmitting diseases. The method used at present relies on the blastomere or trophectoderm biopsy at different development stage [2], which can be associated with a small but significant risk of embryo's damage [3,4]. In this work, we investigated the presence and amplifiability of gDNA in spent media at day 3 and day 5/6, and its suitability for genotyping along with DNA present in embryo blastocoele fluid, in the attempt to test noninvasive or low-invasive matrices as a source of embryo DNA.

In our culture conditions, we found amplifiable gDNA in 93.7% and 94.4% of spent media on day 3 and day 5/6, respectively (Table 1). We also attempted to estimate the quantity of DNA in these samples, despite the variability due to the use of a multicopy gene such as *TBC1D3*. The gDNA amount calculated using *TBC1D3* was not significantly different between the two culture media. The median values were also similar (58 and 67 pg) and 6–7-times higher as compared with DNA amount estimated in blastocoele fluid samples using a similar approach [5]. This fact, although the inhibitory effect of the medium on PCR (see Figure 1), could account for the higher detection rate of *TBC1D3* amplification in medium samples as compared with blastocoele fluid samples reported previously. The gDNA was detected from medium at day 3 in 93.7% of samples analyzed against 63% reported by Stigliani *et al*. [8]. This was probably because of low culture medium volume used in this work, the use of multicopy gene as PCR target and lack of DNA purification procedure. Moreover, new information have been provided on spent medium at day 5/6, which appeared to be more representative of the entire genome, at least for sex determination. In fact, it is noteworthy that the *TSPY1* target was scarcely amplified in day 3 medium samples, as shown by experiments on different media of the same embryos (Table 2), evidencing that gDNA in medium at day 5/6 appears to be more reliable for sex determination. In fact, *TSPY1* results in seven samples of medium at day 5/6 was confirmed during pregnancies or in trophectoderm embryo biopsies. Since the amount of gDNA in day 3 and day 5/6 media was not significantly different, further investigations are needed to explain this fact.

In light of these results, we investigated the DNA in spent medium at day 5/6, along with DNA present in embryo blastocoele fluid, in the attempt to test the suitability for genotyping potential of DNA from these noninvasive matrices. The genotyping of the well-known *MTHFR* C677T polymorphism was chosen essentially for the frequency of 677T allele in the Italian population which was reported to be over 50% in central Italy [12,13], and the availability of IVF patients carrying the 677T allele in homozygosis or heterozygosis, since this mutation has been associated to unexplained recurrent pregnancy loss [14]. WGA followed by PCR with *MTHFR* specific primers and direct sequencing was performed in eight medium and nine blastocoele fluid samples. The C677T polymorphism detection rate was 62.5 and 44.4% in medium and blastocoele fluid, respectively. When both noninvasive samples and biopsy results were available, we found a complete results agreement in one case (embryo 3IB) and a possible case of allele dropout (ADO) in the medium sample of another case (embryo 1IB). ADO, the most prevalent source of genotyping error, is the result of the preferential amplification by chance of one of the two alleles present in a diploid cell. This phenomenon is common in cases where the template DNA is at very low concentrations. Moreover, the WGA step itself could introduce a further variability due to possible preferential amplification of some genomic region [15]. The estimation of the incidence of allele dropout was not possible at this stage due to the lack of adequate reference samples (e.g., the entire embryo) and low sample number. The polymorphism detection rate was low in medium samples and even lower in blastocoele fluid samples, therefore the effective utility of noninvasive matrices for PGD purposes will need to be further investigated in a higher number of samples and/or using methods able to detect not uniformly amplified heterozygous loci or methods with lower detection limits, such as pyrosequencing/next generation sequencing [16] or digital PCR [17], respectively. Notably, the development of a noninvasive medium-based PGD test for α-thalassemias-SEA was recently published by Wu *et al*. [18], corroborating the potential for noninvasive approaches.

## Conclusion

In conclusion, the obtained results highlighted the possibility to easily amplify multicopy genes from cell-free gDNA present in medium at day 5/6 and the possibility, although with limitations, to perform embryo genotyping on a single-copy gene from noninvasive or low-invasive samples such as culture medium at day 5/6 or blastocoele fluid.

## Future perspective

Despite the fact that a multicopy gene was amplified in 94.4% of day 5/6 spent medium samples, still the detection rate of the *MTHFR* polymorphism C677T was too low to account for the use of the extracellular DNA in PGD applications [19]. However, future studies with higher number of samples, the use of molecular methods with lower detection limits (e.g., digital PCR) or the evaluation of different target sequences coupled with linked markers may provide improvement in detection rates, making the extracellular matrices acceptable for PGD applications, therefore avoiding invasive procedures on the embryo. This perspective could be realistic as evidenced by a recently published work focusing on α-thalassemia [18]. Moreover, noninvasive PGD and/or embryo sexing should also be useful in the breeding and dairy industry, allowing fast selection of the preferred animal embryos.

**Table 1.** **Detection and quantification of genomic DNA in spent medium samples at day 3 and day 5/6 through *TBC1D3* amplification.**

**Samples**	**Samples (n)**	***TBC1D3* positive (%)**	**Ct (mean ± SD)**	**Total gDNA content (pg; mean ± SD)**
Spent medium day 3	32	30 (93.7)	31.47 ± 1.67	80 ± 70
Spent medium day 5/6	54	51 (94.4)	30.11 ± 1.78	99 ± 113

Ct: Threshold cycle; gDNA: Genomic DNA; SD: Standard deviation.

**Table 2.** ***TBC1D3* and/or *TSPY1* amplification from different matrices of the same embryos.**

**Embryo ID**	**Medium day 3**		**Medium day 5/6**		**Trophectoderm biopsy**
	***TBC1D3***	***TSPY1***	***TBC1D3***	***TSPY1***	***TSPY1***
1_1	+	–	+	–	NA
2_1	+	–	+	–	NA
3_1	+	–	+	+	NA
4_1	+	–	+	–	NA
5_1	+	–	+	–	NA
6_1	+	–	+	+	NA
1_2	–	–	+	+	NA
2_2	–	–	+	–	NA
3_2	+	–	+	–	NA
4_2	+	–	+	–	NA
5_2	+	–	+	–	NA
6_2	+	–	–	–	NA
7_2	+	–	+	–	NA
B1	+	+	+	+	+
B4	+	–	+	+	+
B8	+	–	+	–	NA

‘+’ and ‘–’ represent positive and negative results, respectively.

NA: Not available.

**Table 3.** ***MTHFR* C677T genotyping results from medium samples.**

**Embryo ID**	**Sample amplified by WGA**	***MTHFR* amplification**	**C677T genotype**
1IB	Day 5/6 medium	+	C
	Trophectoderm biopsy	+	C/T
2IB	Day 5/6 medium	+	C
3IB	Day 5/6 medium	+	C/T
	Trophectoderm biopsy	+	C/T
4IB	Day 5/6 medium	–	NA
	Trophectoderm biopsy	+	T
3	Day 5/6 medium	–	NA
5	Day 5/6 medium	–	NA
6	Day 5/6 medium	+	C
7	Day 5/6 medium	+	C/T

NA: Not available; WGA: Whole-genome amplification.

**Table 4.** ***MTHFR* C677T genotyping results from blastocoele fluid samples.**

**Embryo ID**	**Sample amplified by WGA**	***MTHFR* amplification**	**C677T genotype**
1	Blastocoele fluid	–	NA
2	Blastocoele fluid	+	C/T
1IB	Blastocoele fluid	–	NA
	Trophectoderm biopsy	+	C/T
2IB	Blastocoele fluid	–	NA
3IB	Blastocoele fluid	+	C/T
	Trophectoderm biopsy	+	C/T
3	Blastocoele fluid	+	C/T
5	Blastocoele fluid	+	T
6	Blastocoele fluid	–	NA
7	Blastocoele fluid	–	NA

NA: Not available; WGA: Whole-genome amplification.

**Executive summary**

Preimplantation genetic diagnosis currently relies on biopsy of one or few embryo cells.
Genomic DNA has been reported in the blastocoele fluid and in day 3 embryo culture media.
Using multicopy genes as target and optimizing embryo culture conditions, we amplified target sequences by qPCR in more than 90% of medium samples at day 3 and day 5/6, and we estimated the genomic DNA amount in these samples.
Medium samples at day 5/6 resulted more informative, as shown by amplification of target sequence on Y chromosome.
The possibility to genotype the *MTHFR* polymorphism C677T from media at day 5/6 (n = 8) and blastocoele fluid samples (n = 9) was evaluated by whole-genome amplification, PCR with *MTHFR*-specific primers and direct sequencing of the products.
The C677T polymorphism detection rate was 62.5 and 44.4% in medium and fluid, respectively.
A possible noninvasive approach for embryo genotyping was evidenced, but still with limitations due to low number of samples, low detection rate and possible allele dropout.
